# Characteristics of CD103^+^CD8^+^ T cells in the spleen of *Plasmodium yoelii* NSM-infected mice

**DOI:** 10.3389/fcimb.2025.1668438

**Published:** 2025-12-11

**Authors:** Xingfei Pan, Feihu Shi, Shanni Tang, Meilin Liu, Li Pan, Guikuan Liang, Lu Li, Hongyan Xie, Shan Zhao, Jun Huang

**Affiliations:** 1 Department of Infectious Diseases, Key Laboratory for Major Obstetric Diseases of Guangdong Province, The Third Affiliated Hospital of Guangzhou Medical University, Guangzhou, China; 2 Key Lab of Immunology, Sino-French Hoffmann Institute, Guangzhou Medical University, Guangzhou, China; 3 Department of Laboratory Medicine, The Sixth Affiliated Hospital of Guangzhou Medical University, Qingyuan People’s Hospital, Qingyuan, China; 4 Guangdong Provincial Key Laboratory of Allergy and Clinical Immunology, The Second Affiliated Hospital of Guangzhou Medical University, Guangzhou, China

**Keywords:** CD103, CD8 T cells, *Plasmodium yoelii*, spleen, *LEF1*

## Abstract

**Background:**

CD8^+^ T cells play a critical role in controlling *Plasmodium* infection. CD103, an integrin composed of αE and β7 subunits, is widely recognized as a cell surface marker for tissue-resident memory T (TRM) cells and tumor-infiltrating lymphocytes (TILs).

**Methods:**

In this study, a *Plasmodium* infection model was constructed by intraperitoneally injecting 10^6^ infected red blood cells (iRBCs) into C57BL/6 mice. CD45^+^ cells in the spleen of naïve and infected mice were sorted and subjected to single-cell RNA sequencing (scRNA-seq). The content, activation, and function of CD103^+^CD8^+^ T cells were detected using flow cytometry. qPCR and dual-luciferase reporter assays were performed to find the key transcription factor.

**Results:**

Here, we identified a substantial subset of CD103^+^CD8^+^ T cells in the spleen of naïve mice, whose proportion and count declined rapidly following *Plasmodium yoelii* NSM infection. Compared to CD103^−^CD8^+^ T cells, in both naïve and infected mice, CD103^+^CD8^+^ T cells exhibited higher CD62L expression and lower levels of CD44, CD69, and TIGIT, and they rarely secreted IFN-γ or granzyme B upon PMA plus Ionomycin (PI) stimulation. Single-cell RNA sequencing revealed that differentially expressed genes (DEGs) were enriched in pathways related to “cytoplasmic translation” and “ribosome biosynthesis”, suggesting that these cells are in a pre-activation preparatory state. Bioinformatics predictions and dual-luciferase reporter assays indicated that the transcription factor *LEF1* may regulate *Itgae* transcription by binding to its promoter sequence.

**Conclusions:**

Collectively, our findings demonstrate that splenic CD103^+^CD8^+^ T cells express fewer activation and function-associated molecules, which may contribute to their limited role in the course of *P. yoelii* NSM infection in C57BL/6 mice, and implicates *LEF1* in the regulation of CD103 expression.

## Introduction

As members of the adhesion molecule family, integrins have garnered extensive attention in inflammation, cancer, and immunity (Zarbock et al., 2012). CD103, an integrin composed of αE and β7 subunits, was first identified in the late 1980s for its role in tissue-specific localization of intestinal intraepithelial T cells (Kilshaw and Baker, 1988). Current research on CD103 primarily focuses on T cells and dendritic cells (DCs), where its expression distinguishes a subset of antigen-presenting cells (APCs) with unique functional properties (del Rio et al., 2010).

CD103 is widely studied as a marker for tissue-resident memory T (TRM) cells and tumor-infiltrating lymphocytes (TILs). Memory T cells are classified into subpopulations based on surface marker expression and migration patterns (Schenkel et al., 2014b). TRM cells, characterized by CD69 and CD103 expression, reside at pathogen entry sites and provide rapid first-line defense against infections (Fernandez-Ruiz et al., 2016). In the tumor microenvironment, CD103 binds epithelial E-cadherin to facilitate cell localization—often induced by transforming growth factor β (TGF-β)—and contributes to immune synapse formation between cytotoxic T cells and tumor cells (Le Floc’h et al., 2007). The infiltration of CD103^+^ T cells correlates with better outcomes in epithelial tumors and triple-negative breast cancer (Park et al., 2020). CD103 expression on regulatory T cells (Tregs) has also been reported (Zhang et al., 2019).

T-cell differentiation, whether into effector or memory cells, is regulated by cytokines, metabolites, and transcription factors. For example, homeostatic cytokines IL-7 and IL-15 maintain recirculating memory T cells in secondary lymphoid organs (Surh and Sprent, 2008). TGF-β directly induces CD103 expression via Smad3 or indirectly by counteracting suppression by T-bet (Laidlaw et al., 2014), Eomes (Mackay et al., 2015), and TCF1 (Wu et al., 2020)—factors associated with CD8^+^ terminal effector and central memory differentiation (Teixeiro et al., 2009; Knudson et al., 2017). In TGF-β-rich tumor microenvironments, Smad complexes trigger *Itgae* transcription, leading to CD103 expression (Robinson et al., 2001). However, the characteristics and regulatory mechanisms of CD103^+^CD8^+^ T cells, particularly in the systemic inflammatory context of malaria, remain poorly understood.

Malaria, caused by *Plasmodium* infection, poses a severe global health threat, with an estimated 247 million cases in 2022 (Venkatesan, 2024). *Plasmodium* targets red blood cells (RBCs), with the human life cycle divided into an asymptomatic pre-erythrocytic stage and a symptomatic erythrocytic stage marked by anemia and splenomegaly (Ashley et al., 2018). While malaria vaccines targeting the pre-erythrocytic stage, especially those inducing liver-resident TRM cells, have been explored (Hill, 2006), the changes in CD103 expression in cells during the erythrocytic stage of *Plasmodium* infection remain unclear.

This study investigates the phenotype and characteristics of CD103^+^CD8^+^ T cells in *Plasmodium yoelii*-infected C57BL/6 mice, explores the underlying mechanisms, and identifies upstream regulators of CD103 in CD8^+^ T cells. These findings may enhance understanding of integrin CD103 and clarify the regulatory mechanisms governing CD103^+^CD8^+^ T cells.

## Materials and methods

### Ethical statement

All animal protocols were approved by the Institutional Animal Care and Use Committee of Guangzhou Medical University (S2024-282) and conducted in accordance with institutional guidelines. Every effort was made to minimize animal suffering.

### Mice

Female C57BL/6 wild-type mice (6–8 weeks old) were purchased from Guangdong Zhiyuan Biomedical Technology Co., Ltd. (Guangzhou) and housed under specific pathogen-free conditions at the Animal Center of Guangzhou Medical University.

### Parasites and infection

*P. yoelii* NSM was obtained from the Malaria Research and Reference Reagent Resource Center (MR4). To ensure the vitality of *P. yoelii* and the stability of the experimental results, frozen *P. yoelii* was thawed at 37 °C and resuscitated, and 100 μL was injected intraperitoneally into naïve mice. Tail vein blood smears were prepared daily from days 2 to 5 post-infection and stained with Giemsa (Solarbio, Beijing, China), and parasitemia was quantified. When parasitemia reached 10%–20%, mice were euthanized, and infected RBCs (iRBCs) were counted from aseptically collected ocular blood. Blood was diluted to 10^7^ iRBCs/mL in sterile Phosphate Buffered Saline (PBS), and 100 μL was injected intraperitoneally into each experimental mouse.

### Reagents and antibodies

Roswell Park Memorial Institute (RPMI) 1640, Dulbecco's Modified Eagle Medium (DMEM), fetal bovine serum (FBS), collagenase IV, penicillin, and streptomycin were purchased from Gibco (Invitrogen, Waltham, MA). DNase I was from Solarbio, and RBC lysis buffer (Cat: C3702, 500 mL) was from Beyotime Biotech, Nantong, Jiangsu Province, China. Ampicillin (Cat: MCR003) was obtained from Dingguo Biotech, Beijing, China. Fluorescein-labeled anti-mouse antibodies were sourced from eBiosciences, San Diego, California, BioLegend, San Diego, CA, and Cell Signaling Technology, Danvers, Massachusetts. A detailed list of antibodies used in this study is provided in **Supplementary Table 1**.

### Histology studies

Mice were euthanized 12–16 days post-infection. Livers and spleens from naïve and infected mice were fixed in 10% formalin for 48 hours, paraffin-embedded, sectioned, stained with hematoxylin and eosin (H&E), and examined microscopically.

### Isolation of lymphocytes from tissues

Following euthanasia by 2% isoflurane, peripheral blood was collected in Ethylenediaminetetraacetic acid (EDTA)-containing tubes and diluted 1:1 with PBS. Peripheral blood mononuclear cells (PBMCs) were isolated using Mouse Lymphocyte Isolation Solution (Dakewe, Shenzhen, China) via density gradient centrifugation. Livers, spleens, lungs, tibias, and femurs were harvested. Liver cell suspensions were prepared using the Miltenyi Biotec Liver Isolation Kit, Teterow, Germany, and lymphocytes were isolated via density gradient centrifugation. Lung tissue was minced and digested with 2.4 mg/mL collagenase IV and 0.2 mg/mL DNase I at 37°C for 30 minutes, followed by grinding and filtration. Splenocytes were obtained by grinding spleens using a sterile syringe plunger and filtering through a 100-μm mesh. Bone marrow (BM) cells were flushed from tibias and femurs with RPMI 1640. RBCs in liver, lung, spleen, and BM cell suspensions were lysed using RBC lysis buffer. Isolated cells were washed twice with Hank's Balanced Salt Solution and resuspended in RPMI 1640 containing 10% FBS for subsequent experiments.

### Isolation of iRBCs

When parasitemia reached 20%, peripheral blood was centrifuged to remove serum, resuspended in an equal volume of naïve saline, and subjected to 60%/50% Percoll gradient centrifugation. The iRBC layer was collected, washed to remove Percoll, and resuspended in normal saline, and parasitemia was counted microscopically.

### Flow cytometry analysis

For surface marker detection, single lymphocytes were stained with fluorescence-labeled antibodies against FVD, CD45, CD3, CD19, CD4, CD8, NK1.1, γδT, CD103, CD69, PD-1, CD62L, ICOS, TIGIT, and CD107a at 4°C for 30 minutes. Stained cells were analyzed using flow cytometry (FCM), with data processed using the CytExpert 1.1 software (Beckman Coulter, Brea, CA).

For cytokine (granzyme B, perforin, and IFN-γ) detection, lymphocytes were stimulated with 20 ng/mL phorbol 12-myristate 13-acetate (PMA; Sigma) and 1 μg/mL ionomycin (Sigma, St Louis, MO) at 37°C for 1 hour, followed by the addition of 10 μg/mL brefeldin A (Sigma) to halt cytokine secretion. Cells were stained for surface markers, fixed with 4% paraformaldehyde, permeabilized, and incubated with fluorescence-labeled antibodies for 30 minutes. Stained cells were analyzed using FCM, with data processed using the CytExpert 1.1 software (Beckman Coulter).

For nuclear protein detection, cells were treated with the Invitrogen Foxp3 Transcription Factor Staining Kit and stained with fluorescent antibodies against LEF1, Foxp3, and Ki67 before FCM analysis.

### T-cell proliferation assay

Splenocytes from C57BL/6 mice were resuspended in cold Dulbecco's Phosphate Buffered Saline (DPBS) (0.1% Bovine Serum Albumin (BSA)) at 5 × 10^6^ cells/mL and incubated with 2.5 μM Carboxyfluorescein Succinimidyl Ester (CFSE) for 5 minutes in the dark with agitation. FBS (1/9 volume) was added to quench the reaction, and cells were washed three times with DPBS. The concentration of labeled splenic lymphocytes was adjusted to 2 × 10^6^ cells/mL by RPMI 1640 containing 10% heat-inactivated FBS and stimulated by anti-mouse CD3 (1 μg/mL) plus anti-mouse CD28 (1 μg/mL) for 72 hours in a 37°C CO_2_ incubator. Cells were collected and washed twice with PBS and then stained with anti-mouse CD4-PerCP5.5, anti-mouse CD8-APC-cy7, anti-mouse CD103-APC, and FVD-PB450 for 30 minutes at 4°C in the dark. Stained cells were washed twice with PBS and then analyzed using FCM, with data processed using the CytExpert 1.1 software (Beckman Coulter).

### Cell culture

Human embryonic kidney (HEK) 293T cells were cultured in DMEM with 10% heat-inactivated FBS at 37°C in a 5% CO_2_ incubator.

### Magnetic bead sorting

Lymphocytes were resuspended in sorting buffer and centrifuged, and the supernatant was discarded. Cells were incubated with magnetic bead-conjugated antibodies in sorting buffer at 4°C for 30 minutes. Separation columns were pre-rinsed with sorting buffer, and antibody-labeled cells were loaded onto columns, followed by two washes with sorting buffer. Target cells were eluted after removing the magnetic field.

### Quantitative real-time PCR

Cells were lysed with TRIzol (Invitrogen, Waltham, MA) for RNA extraction. Quantitative PCR (qPCR) was performed using a CFX96 System (Bio-Rad, Hercules, CA). Primer sequences are listed in **Supplementary Table 2**. *β-actin* is the housekeeping gene.

### Single-cell RNA sequencing and bioinformatics analysis

Splenocytes from naïve and infected mice (three each) were obtained according to the protocol mentioned above. Cells from the same group were pooled and mixed, and CD45^+^ cells were sorted using Fluorescence-Activated Cell Sorting (FACS) (Beckman MoFlo). After the viability detection and cell count, the RNA expression profile in each cell was detected using the 10x Genomics Chromium Single Cell 3’s platform in Yuanxin Biotechnology Company (Guangzhou, China). The single cell was barcoded and underwent reverse transcription in oil droplets for the preparation of the cDNA library by the Chromium Single Cell 3′Library and Gel Bead Kit v3. Libraries were sequenced on eight lanes of Illumina NovaSeq 6000 using 150-bp paired-end reads. All data processing and analysis were performed in YUANXIN Biotechnology Co., Ltd, Guangzhou, China. Single-cell RNA sequencing (scRNA-seq) data are available at NCBI under accession numbers SRR22462456 and SRR22476069. Cell Ranger (version 3.1.0) was used to align reads on the GRCm38 reference genome for mouse and generated unique molecular identifier gene expression profiles for every single cell under a standard sequencing quality threshold (Li et al., 2023). The cells were initially divided into 18 clusters by auto-annotation and then manually adjusted to 12 clusters according to the shape of the cell group and the classic gene it transcribes. The heatmap of the top 10 genes in different clusters is shown in **Supplementary Figure 1**, and the numbers of cells detected in each population are listed in **Supplementary Table 3** (<0.2% RBC was mixed in the course of cell sorting and formed an independent cluster). Loupe Brower 6 was used to compare the gene transcription in different cell populations.

### Plasmid construction

PGL3-basic plasmids containing the *Itgae* promoter and luciferase, pCDNA3.1(+) plasmids expressing *Lef1*, and empty vectors were constructed by Tsing Ke Bio-Tech (Beijing, China). Plasmid sequences were verified by enzymatic digestion and sequencing. Prof. Ming-Sheng Cai kindly offered the pRL-TK plasmid.

### Plasmid transfection and dual-luciferase reporter assays

HEK 293T cells were seeded in 24-well plates at 1 × 10^5^ cells/well and cultured for 24 hours. When confluency reached 70%–80%, cells were transfected with plasmids using Lipofectamine 3000 (Invitrogen, Cat: L3000015, Waltham, MA) for 48 hours. Cells were lysed, and luciferase activity was measured using the Dual-Luciferase Reporter Assay System.

### Statistical analysis

Comparisons between two groups were performed using unpaired two-tailed Student’s *t*-tests. Multiple comparisons were analyzed using one-way analysis of variance (ANOVA) followed by Dunnett’s or Tukey’s *post-hoc* tests. Non-parametric data were analyzed using the Kruskal–Wallis or Mann–Whitney U tests with Dunn’s *post-hoc* test. A *p*-value < 0.05 was considered statistically significant.

## Results

### Concentrated expression of CD103 molecules on CD8^+^ T cells in different tissues

**Figure 1:**
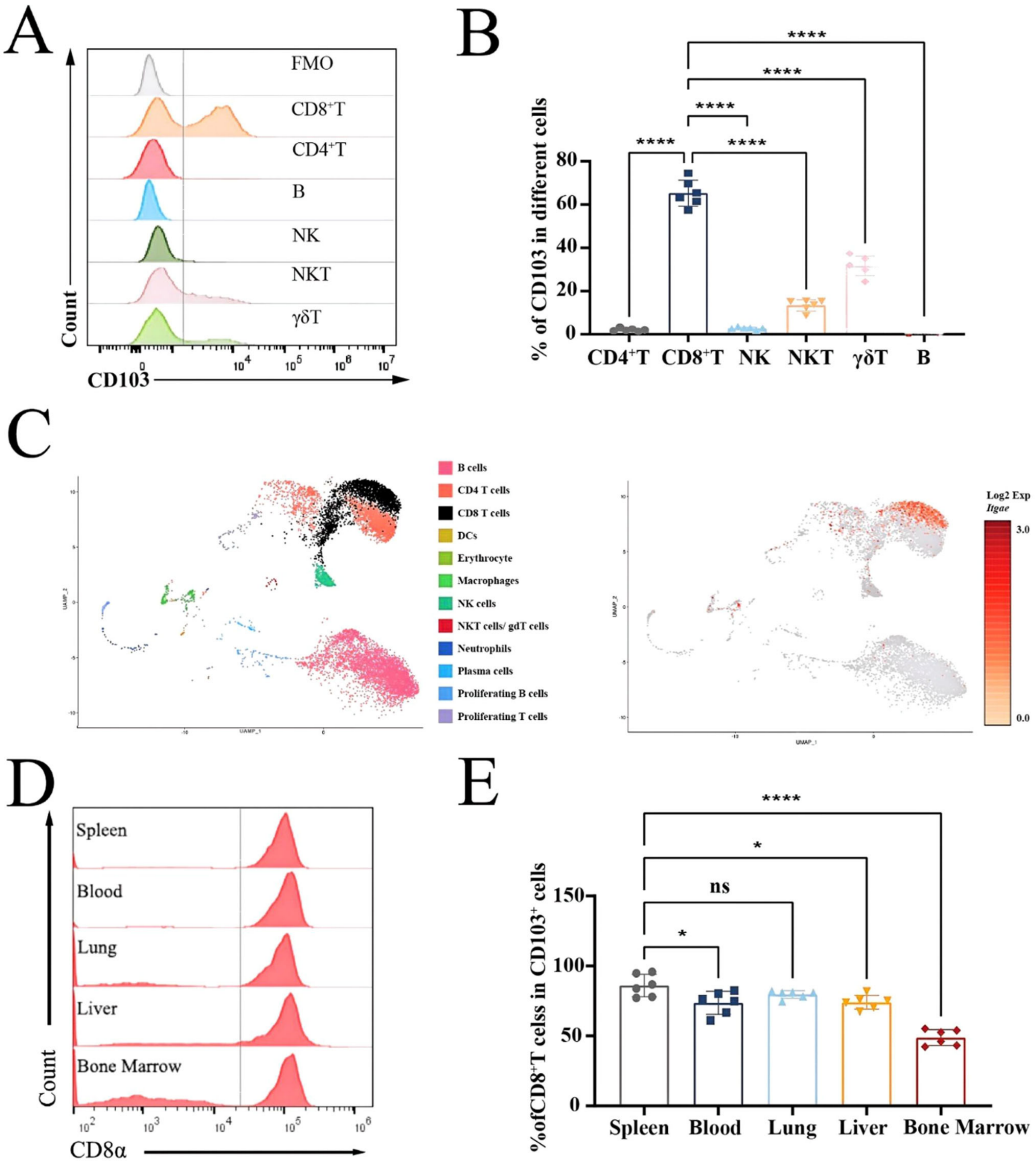
High expression of integrin CD103 on CD8^+^ T cells in different tissues of naïve mice. Spleens from naïve WT C57BL/6 mice were processed into single-cell suspensions. **(A, B)** Flow cytometry was used to detect and quantify CD103 expression on distinct lymphocyte populations. **(C)** Single-cell RNA sequencing of splenic CD45^+^ cells identified 12 annotated cell types. Uniform Manifold Approximation and Projection (UMAP) results showed the location of different cell populations in colors (left) and the density of *Itgae* gene transcription in various cells (right). Each point represents a cell. The log_2_ fold change (log_2_FC) corresponding to different staining is a standardized value (0–3); gray indicates values below 0. **(D, E)** Lymphocytes isolated from peripheral blood, spleen, lung, liver, and bone marrow of naïve mice were analyzed for CD8 expression within CD103^+^ cells, with statistical quantification. Data represent two replicate experiments (three to five mice per group), shown as mean ± SEM. Statistical significance: Student’s *t*-test (*ns p* > 0.05, *p < 0.05, ****p < 0.0001).

To investigate the distribution of the integrin CD103 in lymphocytes, splenocytes from naïve C57BL/6 mice were analyzed using flow cytometry and single-cell RNA sequencing. Based on the gating strategy (**Supplementary Figure S2**), varying degrees of integrin CD103 expression were observed in splenic lymphocytes of naïve mice via FACS (**Figure 1A**). While a CD103^+^ subset was also detected in γδT cells, CD8^+^CD103^+^ T cells were more prominent, accounting for approximately 65.2% of CD8^+^ T cells (**Figure 1B**). At the same time, splenocytes were isolated from naïve mice, and FACS was performed to isolate CD45^+^ cells. scRNA-seq was performed to detect the expression of the *Itgae* gene (which encodes CD103) in different cell populations (**Figure 1C**). The results indicated that the transcription of the *Itgae* gene was primarily concentrated in the CD8^+^ T-cell population (**Figure 1C**). This implied that CD8^+^ T cells were the dominant subset within the CD103^+^ cell population. Additionally, lymphocytes isolated from the peripheral blood, spleen, lung, liver, and bone marrow of naïve C57BL/6 mice were analyzed using flow cytometry. CD103^+^ cells were gated first, and the expression of CD8a was counted (**Figure 1D**). As shown in **Figure 1E**, the results showed that approximately 80% of CD103-expressing cells in the spleen of naïve mice were CD8^+^ T cells, and a similar percentage was found in the lung, which was higher than that in the peripheral blood, liver, and bone marrow. Altogether, these results indicated that the CD103 molecule was mainly expressed on CD8^+^ T cells in different tissues from naïve mice.

### *P. yoelii* NSM infection reduces CD103 expression on splenic CD8^+^ T cells

**Figure 2:**
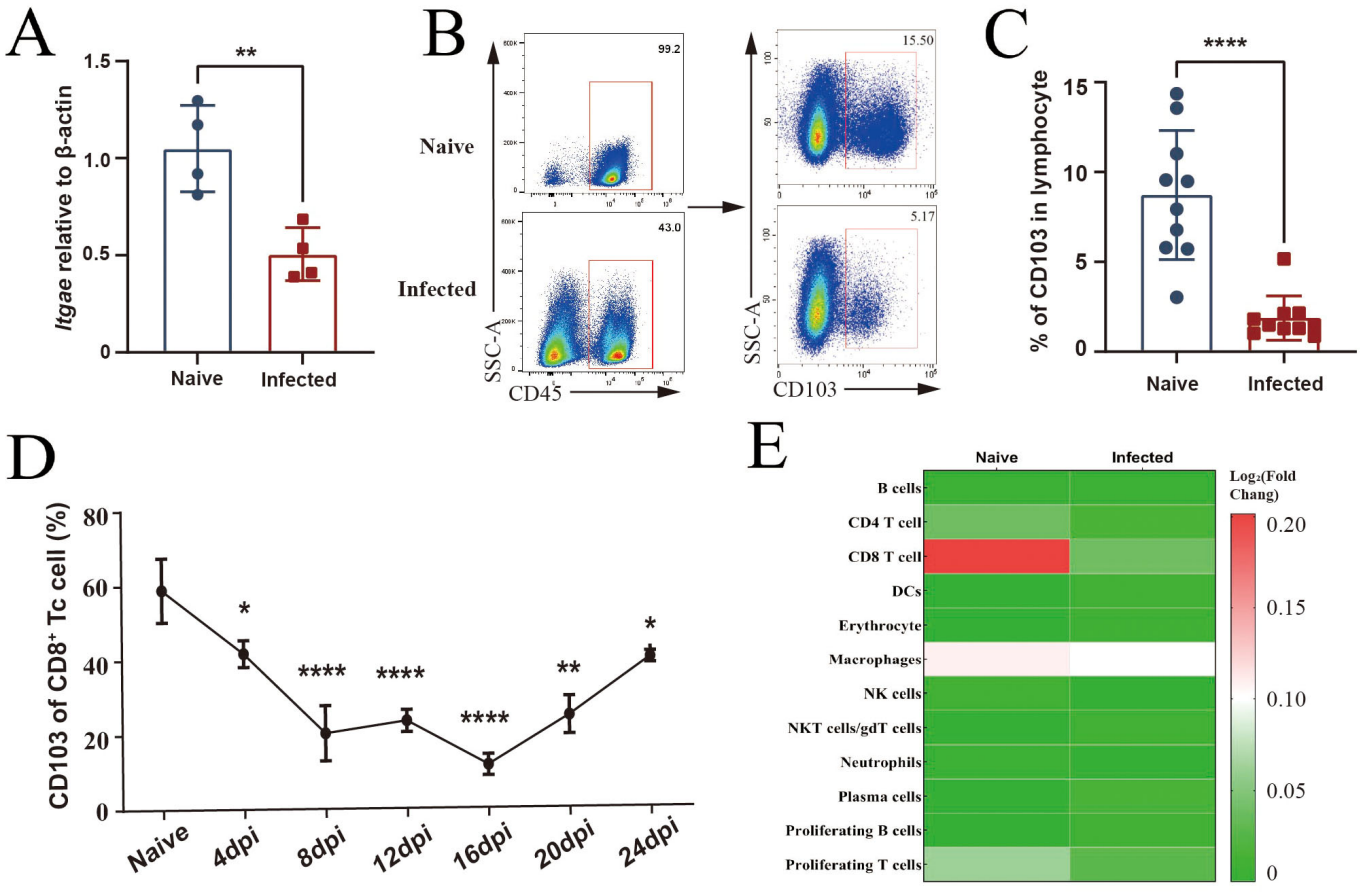
*Plasmodium yoelii* NSM-infection reduces CD103 expression on splenic CD8^+^ T cells. **(A)** qRT-PCR measured CD103 transcriptional levels in splenic lymphocytes from naïve *vs*. infected mice. *β-actin* is the housekeeping gene. **(B, C)** Flow cytometry analyzed CD103 expression on CD45^+^ lymphocytes in the spleen. **(D)** Dynamic changes in CD103 expression on the splenic CD8^+^ cells from *P. yoelii* NSM-infected mice were detected from 0 to 24 dpi, with a 4-day interval. **(E)** Single-cell RNA sequencing on the spleen cells of naïve and 12-dpi mice, revealing changes in the transcription intensity of the gene *Itgae* across different cell populations of the spleen before and after infection. The log_2_ fold change (log_2_FC) corresponding to different staining is a standardized value. Data from three replicate experiments (three to five mice per group) are shown as mean ± SEM. Statistical significance: Student’s *t*-test (*ns p* > 0.05, *p < 0.05, **p < 0.01, ***p < 0.001). dpi, days post-infection.

*P. yoelii* NSM infection could destroy RBC and induce inflammatory changes in the spleen of C57BL/6 mice, especially on 12–16 days post-infection (dpi) (**Supplementary Figure S3**). To explore the change of CD103 expression in CD8^+^ T cells in the course of *P. yoelii* NSM infection, splenic lymphocyte suspensions were prepared from both naïve and infected mice. RNA was extracted and reverse-transcribed to cDNA, and *Itgae* transcription was detected using qPCR. As shown in **Figure 2A**, *Itgae* transcription in the spleens of infected mice was significantly decreased (*p* < 0.05). At the same time, lymphocytes were stained with different fluorescence-labeled antibodies to detect the expression of CD103 on CD45^+^ T cells, too. Flow cytometry analysis confirmed a reduction in the percentage of CD103^+^ cells within the CD45^+^ lymphocyte population in infected mice (*p* < 0.05, **Figures 2B, C**). Further examination revealed that the proportion of CD103 on CD8^+^ T cells continued to decrease from 4 to 16 dpi, and then the percentage increased from 16 to 24 dpi (**Figure 2D**). A significant reduction in the rate of CD103-expressing CD8^+^ T cells was also observed in the blood, liver, lung, and bone marrow 12–16 days after infection (*p* < 0.05, **Supplementary Figure S4A**). Longitudinal analysis of CD103 expression on blood CD8^+^ T cells supported this result, too (*p* < 0.05, **Supplementary Figure S4B**). Single-cell RNA sequencing of FACS-sorted splenic CD45^+^ cells was used to detect the distribution of *Itgae* in different cell populations. The results showed that *Itgae* transcription was notable in CD8^+^ T cells from naïve mice. However, The level of IItgae transcription in CD8+ T cells from infected mice was decreased compared to naive mice (**Figure 2E**). These results indicated that *P. yoelii* NSM infection could reduce CD103 expression on splenic CD8^+^ T cells in mice.

### Splenic CD103^+^CD8^+^ T cells exhibit naïve-like properties after *P. yoelii* NSM infection

**Figure 3:**
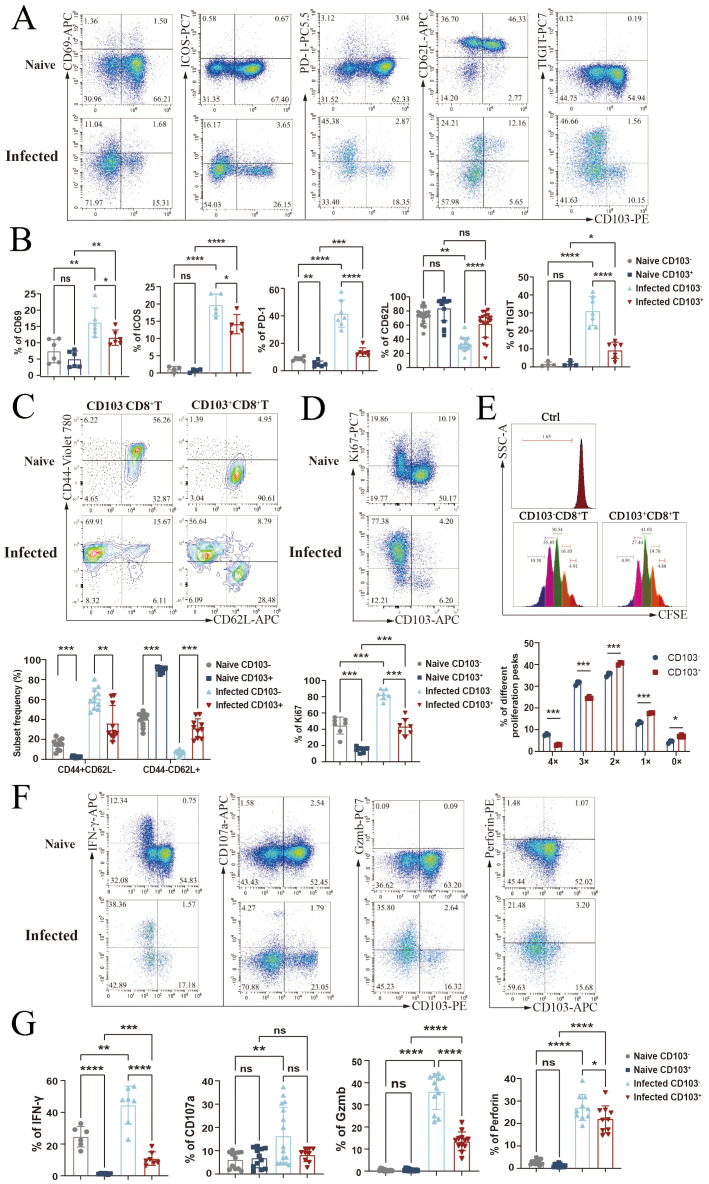
Naïve-like properties of CD8^+^CD103^+^ T cells. Splenic single-cell suspensions from naïve and infected mice were analyzed. **(A, B)** Flow cytometry quantified co-expression of CD103 with activation markers (CD69, ICOS, and CD62L) and inhibitory molecules (PD-1 and TIGIT) on CD8^+^ T cells. **(C)** Effector (CD44^+^CD62L^−^) *vs*. resting (CD44^−^CD62L^+^) subsets within CD103^+^CD8^+^*vs*. CD103^−^CD8^+^ populations. **(D)** CD103/Ki67 co-expression on CD8^+^ T cells pre- and post-infection. **(E)** CFSE-labeled naïve splenocytes were stimulated with anti-CD3/CD28; 3-day proliferation of CD103^+^*vs*. CD103^−^CD8^+^ T cells was measured. **(F, G)** Co-expression of CD103 with cytokines (IFN-γ, CD107a, granzyme B, and perforin) on CD8^+^ T cells from both naïve and infected mice. Data represent two to three independent experiments (three to five mice per group), shown as mean ± SEM. Statistical significance: Student’s *t*-test (*ns p* > 0.05, *p < 0.05, **p < 0.01, ***p < 0.001, ****p < 0.0001).

To characterize the phenotypes of CD103^+^CD8^+^ T cells before and after infection, single splenocytes were stained with fluorescent antibodies against surface markers. In naïve mice, CD103^−^CD8^+^ T cells and CD103^+^CD8^+^ T cells showed no significant differences in the expression of CD69, ICOS, CD62L, or TIGIT (*p* > 0.05). However, after infection, CD103^−^CD8^+^ T cells were activated, with upregulated expression of CD69, ICOS, PD-1, and TIGIT (*p* < 0.05). While CD103^+^ cells also showed increased CD69 and ICOS expression post-infection, the changes were less pronounced than in CD103^−^ cells. These results indicate that infection enhances the activation of CD103^−^CD8^+^ T cells, whereas CD103^+^CD8^+^ T cells retain more resting characteristics (**Figures 3A, B**).

Moreover, flow cytometry analysis of effector and resting subsets in CD103^+/−^CD8^+^ T cells post-infection (**Figure 3C**) revealed that CD103^−^CD8^+^ T cells contained a higher proportion of CD44^hi^CD62L^low^ effector subsets, while CD103^+^CD8^+^ T cells were predominantly in the CD44^low^CD62L^hi^ resting subset (*p* < 0.05).

Comparison of cell proliferation capacity by flow cytometry showed a higher percentage of Ki67^+^ cells in CD103^−^CD8^+^ T cells from infected mice (*p* < 0.05), indicating stronger proliferative ability compared to CD103^+^CD8^+^ T cells (*p* < 0.05, **Figure 3D**). *In vitro* stimulation of CFSE-stained naïve splenocytes with CD3 and CD28 mAbs was performed for 3 days; cells were washed twice and stained with different fluorescent-labeled antibodies for mouse CD4, CD8, CD103, and FVD. FACS results showed that CD103^−^CD8^+^ T-cell proliferation peaks were concentrated at 4× and 3×, while those of CD103^+^ cells were at 2× and 1×, suggesting delayed proliferation in CD103^+^CD8^+^ T cells (**Figure 3E**).

Additionally, flow cytometry detection of function-associated molecules showed increased percentages of CD107a, IFN-γ, granzyme B, and perforin-expressing cells in CD8^+^ T cells from infected mice (*p* < 0.05). However, most of these cytotoxic molecules were expressed in CD103^−^CD8^+^ T cells rather than CD103^+^CD8^+^ T cells, particularly in infected mice (**Figures 3F, G**). These results suggested that CD103^−^CD8^+^ T cells, rather than CD103^+^CD8^+^ T cells, contribute to the antimalarial immune response.

### Splenic *Itgae*^+^CD8^+^ T cells transcribe fewer inflammation and activation-related genes after infection

**Figure 4:**
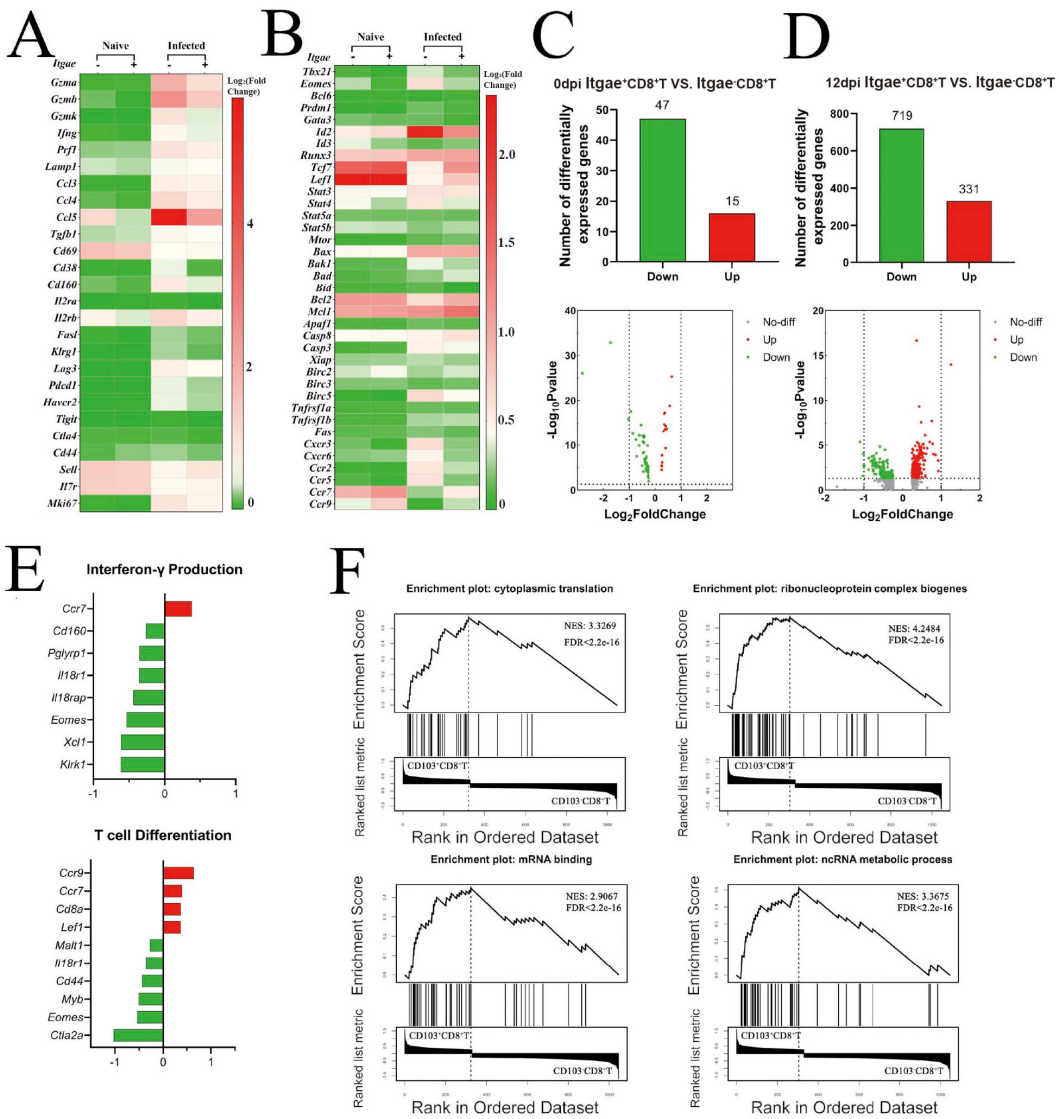
*Itgae*^+^CD8^+^ splenic T cells transcribe fewer inflammation and activation-related genes after infection. Single-cell RNA sequencing was performed on splenocytes from naïve and infected mice. **(A, B)** CD8^+^ T-cell populations from both naïve and 12-dpi mice were selected, classified according to the expression of *Itgae* (*Itgae*^+^*vs*. *Itgae*^−^) to compare transcription of cytotoxic molecules, activation/inhibition markers, differentiation-related transcription factors, apoptosis proteins, and chemokine receptors. The log_2_ fold change (log_2_FC) corresponding to different staining is a standardized value. **(C, D)** The number of genes with differential transcription DEGs is shown; *Itgae*^+^CD108^+^ cells compared to *Itgae*^−^CD8^+^ cells from naïve and 12-dpi mice. **(E)** Horizontal bar chart of enriched DEG expression changes in “interferon-γ production” and “T-cell differentiation” pathways. **(F)** GSEA of differential genes in the infected group. dpi, days post-infection; DEGs, differentially expressed genes; GSEA, Gene Set Enrichment Analysis.

Next, the RNA transcription differences in CD8^+^ T cells from naïve and infected mice were compared, both with and without the Itgae gene transcribed, using scRNA-seq. The transcription of function-, activation-, suppression-, and proliferation-related genes between *Itgae*^−^CD8^+^ and *Itgae*^+^CD8^+^ T cells, in both naïve and infected, was compared (**Figures 4A, B**). The results showed that most pro-inflammatory markers, such as granzyme, Lamp1, Ifng, Prf1, Ccl5, Emoes, and Id2 transcription, increased after infection. However, compared to that in CD103^+^CD8^+^ T cells, the gene transcription is more active in CD103^−^CD8^+^ T cells. At the same time, the transcription of genes related to naïve or memory T cells, such as *Sell*, *Il7r*, *Tcf7*, and *Lef1*, decreased after infection. Compared to *Itgae*^+^CD8^+^ T cells, the reduction of this gene transcription in *Itgae*^−^CD8^+^ T cells in infected mice is more obvious. These results are consistent with the report that *Itgae*^−^CD8^+^ T cells may be a population of effector cells. In contrast, reduced function, as observed using FACS, suggests that Itgae^−^CD8^+^ T cells could represent a population of effector cells with diminished functionality.

Moreover, the number of differentially expressed genes (DEGs) between the *Itgae*^+^CD8^+^ and Itgae^−^CD8^+^ T cells in naïve and infected mice was compared. A total of 62 DEGs (47 downregulated and 15 upregulated) were found in naïve mice (**Figure 4C**), while 1,050 DEGs (719 downregulated and 331 upregulated) were found in infected mice (**Figure 4D**). These results indicated that noticeable differences exist in gene transcription between *Itgae*^+^CD8^+^ and *Itgae*^−^CD8^+^ T cells, especially in the infected mice. It supports the suggestion that the CD103^+^CD8^+^ T cell population is different from the CD103^−^CD8^+^ T cells.

Furthermore, DEGs between *Itgae*^+^CD8^+^ T cells and *Itgae*^−^CD8^+^ T cells from both naïve mice and 12-dpi mice were subjected to Gene Ontology (GO) and Kyoto Encyclopedia of Genes and Genomes (KEGG) enrichment analyses; the enriched gene pathways are shown in **Supplementary Figures S5A–D**. “Interferon-γ production” and “T-cell differentiation” pathways were enriched between *Itgae*^+^CD8^+^ T cells and *Itgae*^−^CD8^+^ T cells from infected mice. DEGs in these two pathways were listed, and the related contents were compared. As shown in **Figure 4E**, most of the interferon-γ production-related genes, such as *Cd160*, *Pglyrp1*, *Il18r1*, *Il18rap*, *Eomes*, *Xcl1*, and *Klrk1*, were downregulated in *Itgae*^+^CD8^+^ T cells compared to *Itgae*^−^CD8^+^ T cells. It is consistent with the FACS results, which showed that most of the IFN-γ expression was secreted by CD103^−^CD8^+^ T cells (**Figure 3F**). Similar results were found in DEGs in “T-cell differentiation”, “cytokine–cytokine receptor interaction”, “cytokine binding”, “leukocyte cell–cell adhesion”, and “regulation of immune effector process” pathway (**Supplementary Figure S5E**).

At the same time, Gene Set Enrichment Analysis (GSEA) of DEGs in the infected group showed high scores for “cytoplasmic translation”, “ribonucleoprotein complex biogenesis”, “mRNA binding”, and “ncRNA metabolic process” (**Figure 4F**), indicating active biological processes in *Itgae*^+^CD8^+^ T cells. Conversely, the chemokine signaling pathway and cytokine–cytokine receptor interaction gene sets showed negative enrichment (**Supplementary Figure S5F**). Altogether, these results indicated that *Itgae*^+^CD8^+^ T cells may be in a pre-mobilization state despite exhibiting some practical function.

### *Lef1* could bind to the CD103 promoter

**Figure 5:**
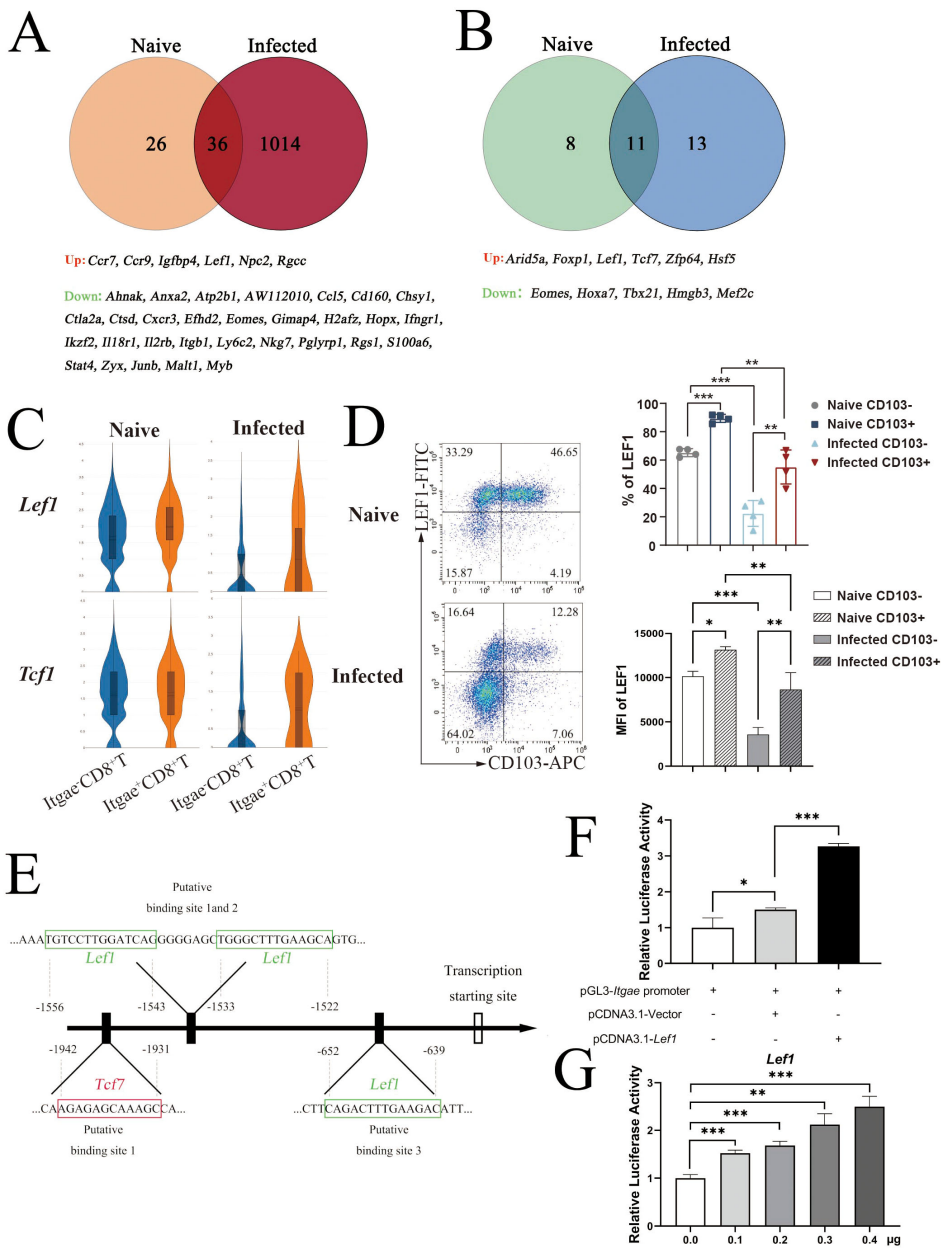
Transcription factor LEF1 binds to the *Itgae* promoter and regulates its transcription. Differentially expressed genes (DEGs) between *Itgae*^+^CD8^+^ and *Itgae*^−^CD8^+^ T cells were collected from single-cell RNA sequencing results of both naïve and infected mice (12 dpi). **(A)** These results were overlapped, and the overlapped genes are listed at the bottom. **(B)** Transcription factor profiles of *Itgae*^+^*vs*. *Itgae*^−^CD8^+^ T cells from naïve and infected mice were collected via single-cell RNA sequencing, and the results were compared. Similarly, *Itgae*^+^*vs*. *Itgae*^−^CD8^+^ T cells from naïve and infected mice were collected, and their results were compared. The overlapped genes were identified and listed at the bottom. **(C)** Violin plots showing *LEF1* and *TCF1* transcription in both *Itgae*^+^ and *Itgae*^−^CD8^+^ T cell subsets under naïve and infected conditions. **(D)** Flow cytometry analysis of CD103 and *LEF1* co-expression on CD8^+^ T cells from both naïve and infected mice. Both the percentage and median fluorescence intensity of *LEF1* transcription were measured. **(E)** JASPAR database prediction of *LEF1*/*TCF1* binding sites in the *Itgae* promoter. **(F, G)** HEK 293T cells co-transfected with pGL3-*Itgae* promoter and pCDNA3.1-*LEF1* (or empty vector) were assayed for relative fluorescence; dose-dependent effects of *LEF1* were evaluated. Data from three independent experiments are shown as mean ± SEM. Statistical significance: Student’s *t*-test (*ns p* > 0.05, *p < 0.05, **p < 0.01, ***p < 0.001, ****p < 0.0001). dpi, days post-infection.

To identify genes regulating CD103, DEGs between CD103^+^CD8^+^ and CD103^−^CD8^+^ T cells from naïve and infected mice were intersected, and a Venn diagram was generated (**Figure 5A**). Lymphocyte enhancer-binding factor 1 (*Lef1*, encoded by *Lef1*) showed significant differences in both groups. *Lef1*, a high-mobility group (HMG) family transcription factor, is a homolog of T-cell factor 1 (TCF1, encoded by *Tcf7*). Transcription factor analysis of sequencing data revealed the differential transcription of *Tcf1* and *Lef1* between the two groups (**Figure 5B**). Although *Lef1* transcription decreased after infection, it remained higher in *Itgae*^+^CD8^+^ T cells than in *Itgae*^−^CD8^+^ T cells (**Figure 5C**). Flow cytometry confirmed high LEF1 expression on CD103^+^CD8^+^ T cells in both naïve and infected states (**Figure 5D**).

To verify *Lef1*-mediated regulation of *Itgae* transcription, potential *Lef1* and Tcf1 binding sites on the CD103 promoter were predicted using JASPAR (**Figure 5E**). *Lef1* was predicted to have three binding sites in the region from −2,000 bp upstream to 100 bp downstream of the CD103 transcription start site, while *Tcf1* had only one. Dual-luciferase reporter assays were performed by co-transfecting 293T cells with a pGL3 plasmid containing the CD103 promoter and luciferase gene, a pCDNA3.1(+) plasmid expressing *Lef1* (or empty vector), and the pRL-TK plasmid (**Figure 5F**). *Lef1* transfection strongly induced CD103 promoter activity compared to the empty vector. Dose-dependent co-transfection of *Lef1*-expressing plasmids with the CD103 promoter luciferase reporter vector in 293T cells confirmed the specific activation of CD103 promoter activity by *Lef1* (**Figure 5G**).

## Discussion

The skin and mucous membranes are the body’s first line of defense against external pathogen infections. CD8^+^ TRM cells have attracted significant attention owing to their crucial role in pathogen clearance, vaccination, and tumor immunity (Mackay et al., 2013; Schenkel et al., 2014a). As a marker of CD8^+^ TRM cells, CD103 promotes the migration and long-term retention of these cells in tissues (Hardenberg et al., 2018). In addition to CD8^+^ T cells, CD103-expressing DCs (Yang et al., 2024) and NK cells (Santana-Hernández et al., 2024) were identified, and they play vital roles in treating clinical diseases.

Both *Plasmodium vivax* and *Plasmodium falciparum* are human malaria parasites and are the primary pathogens causing malaria worldwide. They can lead to human illness (the former has milder symptoms, such as fever, while the latter is fatal) (Chu et al., 2023). *P. yoelii* is a rodent malaria parasite (its primary host is the mouse, and it does not infect humans; it is only used for laboratory research, such as vaccine development and antimalarial drug testing) (Li et al., 2023). CD8^+^ T cells play a crucial role in parasite clearance and erythrocyte removal in the spleen (Safeukui et al., 2015). During the exo-erythrocytic stage, liver CD8^+^ TRM cells form a frontline defense against malaria liver-stage infection (Fernandez-Ruiz et al., 2016). Therefore, understanding the properties and mechanisms underlying CD103 expression is of great significance for malaria prevention and treatment.

Using flow cytometry (FACS) and single-cell RNA sequencing, our study revealed a large population of CD103-expressing CD8^+^ T cells in naïve mice, with their numbers significantly reduced after *Plasmodium* infection. This phenomenon has been observed across multiple organs, likely due to multi-organ inflammation induced by the infection. Given that CD103 is vital for the tissue retention of T cells (Hardenberg et al., 2018), the downregulation of its expression during infection may enhance the mobility of activated effector CD8^+^ T cells, enabling them to survey the body and clear infected cells. A similar pattern has been reported in mice infected with lymphocytic choriomeningitis virus (Wu et al., 2021). Consistent with this, strong T-cell receptor activation induces the upregulation of T-bet and EOMES, which may inhibit CD103 expression (Sullivan et al., 2003; Kaech and Cui, 2012).

CD44 and CD62L were used to define naïve or memory T cells (Gattinoni et al., 2009). Our results indicated that CD103^+^CD8^+^ T cells were predominantly in the CD44^low^CD62L^hi^ resting subset. Moreover, CD69, ICOS, and CD62L are classic markers associated with T-cell activation (Wikenheiser and Stumhofer, 2016; Cibrián and Sánchez-Madrid, 2017). Upon activation, CD8^+^ T cells express inhibitory receptors such as PD-1 and TIGIT; secrete cytokines, granzyme B, and perforin; and undergo degranulation to participate in immune processes (Aktas et al., 2009; Hojo-Souza et al., 2020). Our results showed that, in both naïve and infected mouse spleens, the CD103^+^CD8^+^ T-cell population expressed higher levels of CD62L and lower levels of both CD69 and TIGIT. It suggested that CD103^+^CD8^+^ T cells may be a population of naïve T cells. It implied that CD103 may serve as a marker for naïve T cells.

Furthermore, CD103^+^CD8^+^ T cells were found to secrete IFN-γ and granzyme B rarely upon PI stimulation, with fewer Ki67^+^ cells, and delayed proliferative ability compared to CD103^−^CD8^+^ T cells. These results suggest that CD103^+^CD8^+^ T cells may be a population of “quiescent cells” in either naïve or infected mice. Single-cell RNA sequencing data corroborated these findings. Additionally, in the single-cell RNA sequencing results, DEGs in the infected group were enriched in gene clusters related to “ribonucleoprotein complex biogenesis” and “mRNA binding”, indicating that CD103^+^CD8^+^ T cells exhibit limited active functions; they may be primed for mobilization.

*Tcf1* and *Lef1* are known to regulate the specification of thymic progenitors to the T-cell lineage, instruct CD4^+^ T-cell lineage choice, and establish CD8^+^ T-cell identity (De Obaldia and Bhandoola, 2015). *Lef1* is essential for stem cell maintenance and organ development, particularly in its role in epithelial–mesenchymal transition (EMT) (Santiago et al., 2017). Aberrant *Lef1* transcription is involved in tumorigenesis, as well as cancer cell proliferation, migration, and invasion (Zirkel et al., 2013; Liang et al., 2015). However, in adult tissues, its expression is tissue-specific, mainly localized to the thymus or T cells (Hrckulak et al., 2016). Our data indicate that CD103 is primarily expressed in CD8^+^ T cells, a finding verified in multiple organs. Consistent with other reports, CD103^+^CD8^+^ T cells are readily detectable in lymphoid and non-lymphoid tissues, as well as in peripheral blood (Sathaliyawala et al., 2013; Woon et al., 2016). It has been reported that *Tcf1* is recruited to the *Itgae* locus and regulates CD103 expression (Wu et al., 2020). In our experiments, *Lef1* was found to bind to the CD103 promoter. This suggests that *Lef1* may be involved in the regulation of CD103 expression in the course of *Plasmodium* infection.

In summary, our study revealed that CD103^+^CD8^+^ T cells may represent a population of “quiescent cells” that do not respond rapidly to stimulation in either naïve or infected mice and suggested that *Lef1* may regulate CD103 expression. These results provide new insights for the study of CD103^+^ T cells.

## Data Availability

The datasets presented in this study can be found in online repositories. The names of the repository/repositories and accession number(s) can be found in the article/**Supplementary Material**.

## References

[B1] Aktas E. Kucuksezer U. C. Bilgic S. Erten G. Deniz G. (2009). Relationship between CD107a expression and cytotoxic activity. Cell Immunol. 254, 149–154. doi: 10.1016/j.cellimm.2008.08.007, PMID: 18835598

[B2] Ashley E. A. Pyae Phyo A. Woodrow C. J. (2018). Malaria. Lancet 391, 1608–1621. doi: 10.1016/S0140-6736(18)30324-6, PMID: 29631781

[B3] Chu C. S. Stolbrink M. Stolady D. Saito M. Beau C. Choun K. . (2023). Severe falciparum and vivax malaria on the Thailand-Myanmar border: A review of 1503 cases. Clin. Infect. Dis. 77, 721–728. doi: 10.1093/cid/ciad262, PMID: 37144342 PMC10495127

[B4] Cibrián D. Sánchez-Madrid F. (2017). CD69: from activation marker to metabolic gatekeeper. Eur. J. Immunol. 47, 946–953. doi: 10.1002/eji.201646837, PMID: 28475283 PMC6485631

[B5] del Rio M. L. Bernhardt G. Rodriguez-Barbosa J. I. Förster R. (2010). Development and functional specialization of CD103+ dendritic cells. Immunol. Rev. 234, 268–281. doi: 10.1111/j.0105-2896.2009.00874.x, PMID: 20193025

[B6] De Obaldia M. E. Bhandoola A. (2015). Transcriptional regulation of innate and adaptive lymphocyte lineages. Annu. Rev. Immunol. 33, 607–642. doi: 10.1146/annurev-immunol-032414-112032, PMID: 25665079

[B7] Fernandez-Ruiz D. Ng W. Y. Holz L. E. Ma J. Z. Zaid A. Wong Y. C. . (2016). Liver-resident memory CD8(+) T cells form a front-line defense against malaria liver-stage infection. Immunity 45, 889–902. doi: 10.1016/j.immuni.2016.08.011, PMID: 27692609

[B8] Gattinoni L. Zhong X. S. Palmer D. C. Ji Y. Hinrichs C. S. Yu Z. . (2009). Wnt signaling arrests effector T cell differentiation and generates CD8+ memory stem cells. Nat. Med. 15, 808–813. doi: 10.1038/nm.1982, PMID: 19525962 PMC2707501

[B9] Hardenberg J. B. Braun A. Schön M. P. (2018). A yin and yang in epithelial immunology: the roles of the α(E)(CD103)β(7) integrin in T cells. J. Invest. Dermatol. 138, 23–31. doi: 10.1016/j.jid.2017.05.026, PMID: 28941625

[B10] Hill A. V. (2006). Pre-erythrocytic malaria vaccines: towards greater efficacy. Nat. Rev. Immunol. 6, 21–32. doi: 10.1038/nri1746, PMID: 16493425

[B11] Hojo-Souza N. S. De Azevedo P. O. De Castro J. T. Teixeira-Carvalho A. Lieberman J. Junqueira C. . (2020). Contributions of IFN-γ and granulysin to the clearance of Plasmodium yoelii blood stage. PLoS Pathog. 16, e1008840. doi: 10.1371/journal.ppat.1008840, PMID: 32913355 PMC7482970

[B12] Hrckulak D. Kolar M. Strnad H. Korinek V. (2016). TCF/LEF transcription factors: an update from the internet resources. Cancers (Basel) 8 (7), 70. doi: 10.3390/cancers8070070, PMID: 27447672 PMC4963812

[B13] Kaech S. M. Cui W. (2012). Transcriptional control of effector and memory CD8+ T cell differentiation. Nat. Rev. Immunol. 12, 749–761. doi: 10.1038/nri3307, PMID: 23080391 PMC4137483

[B14] Kilshaw P. J. Baker K. C . A unique surface antigen on intraepithelial lymphocytes in the mouse. Immunol Lett. (1988) 18 (2), 149–154. doi: 10.1016/0165-2478(88)90056-9, PMID: 3042615 10.1016/0165-2478(88)90056-9

[B15] Knudson K. M. Pritzl C. J. Saxena V. Altman A. Daniels M. A. Teixeiro E. (2017). NFκB-Pim-1-Eomesodermin axis is critical for maintaining CD8 T-cell memory quality. Proc. Natl. Acad. Sci. U.S.A. 114, E1659–e1667. doi: 10.1073/pnas.1608448114, PMID: 28193872 PMC5338529

[B16] Laidlaw B. J. Zhang N. Marshall H. D. Staron M. M. Guan T. Hu Y. . (2014). CD4+ T cell help guides formation of CD103+ lung-resident memory CD8+ T cells during influenza viral infection. Immunity 41, 633–645. doi: 10.1016/j.immuni.2014.09.007, PMID: 25308332 PMC4324721

[B17] Le Floc’h A. Jalil A. Vergnon I. Le Maux Chansac B. Lazar V. Bismuth G. . (2007). Alpha E beta 7 integrin interaction with E-cadherin promotes antitumor CTL activity by triggering lytic granule polarization and exocytosis. J. Exp. Med. 204, 559–570. doi: 10.1084/jem.20061524, PMID: 17325197 PMC2137907

[B18] Li J. Liu L. Xing J. Chen D. Fang C. Mo F. . (2023). TLR7 modulates extramedullary splenic erythropoiesis in P. yoelii NSM-infected mice through the regulation of iron metabolism of macrophages with IFN-γ. Front. Immunol. 14, 1123074. doi: 10.3389/fimmu.2023.1123074, PMID: 37180169 PMC10174296

[B19] Liang J. Li X. Li Y. Wei J. Daniels G. Zhong X. . (2015). LEF1 targeting EMT in prostate cancer invasion is mediated by miR-181a. Am. J. Cancer Res. 5, 1124–1132., PMID: 26045991 PMC4449440

[B20] Mackay L. K. Rahimpour A. Ma J. Z. Collins N. Stock A. T. Hafon M. L. . (2013). The developmental pathway for CD103(+)CD8+ tissue-resident memory T cells of skin. Nat. Immunol. 14, 1294–1301. doi: 10.1038/ni.2744, PMID: 24162776

[B21] Mackay L. K. Wynne-Jones E. Freestone D. Pellicci D. G. Mielke L. A. Newman D. M. . (2015). T-box transcription factors combine with the cytokines TGF-β and IL-15 to control tissue-resident memory T cell fate. Immunity 43, 1101–1111. doi: 10.1016/j.immuni.2015.11.008, PMID: 26682984

[B22] Park M. H. Kwon S. Y. Choi J. E. Gong G. Bae Y. K. (2020). Intratumoral CD103-positive tumour-infiltrating lymphocytes are associated with favourable prognosis in patients with triple-negative breast cancer. Histopathology 77, 560–569. doi: 10.1111/his.14126, PMID: 32333690

[B23] Robinson P. W. Green S. J. Carter C. Coadwell J. Kilshaw P. J. (2001). Studies on transcriptional regulation of the mucosal T-cell integrin alphaEbeta7 (CD103). Immunology 103, 146–154. doi: 10.1046/j.1365-2567.2001.01232.x, PMID: 11412301 PMC1783235

[B24] Safeukui I. Gomez N. D. Adelani A. A. Burte F. Afolabi N. K. Akondy R. . (2015). Malaria induces anemia through CD8+ T cell-dependent parasite clearance and erythrocyte removal in the spleen. mBio 6(1), e02493-14. doi: 10.1128/mBio.02493-14, PMID: 25604792 PMC4324318

[B25] Santana-Hernández S. Suarez-Olmos J. Servitja S. Berenguer-Molins P. Costa-Garcia M. Comerma L. . (2024). NK cell-triggered CCL5/IFNγ-CXCL9/10 axis underlies the clinical efficacy of neoadjuvant anti-HER2 antibodies in breast cancer. J. Exp. Clin. Cancer Res. 43, 10. doi: 10.1186/s13046-023-02918-4, PMID: 38167224 PMC10763072

[B26] Santiago L. Daniels G. Wang D. Deng F. M. Lee P. (2017). Wnt signaling pathway protein LEF1 in cancer, as a biomarker for prognosis and a target for treatment. Am. J. Cancer Res. 7, 1389–1406., PMID: 28670499 PMC5489786

[B27] Sathaliyawala T. Kubota M. Yudanin N. Turner D. Camp P. Thome J. J. . (2013). Distribution and compartmentalization of human circulating and tissue-resident memory T cell subsets. Immunity 38, 187–197. doi: 10.1016/j.immuni.2012.09.020, PMID: 23260195 PMC3557604

[B28] Schenkel J. M. Fraser K. A. Beura L. K. Pauken K. E. Vezys V. Masopust D. (2014a). T cell memory. Resident memory CD8 T cells trigger protective innate and adaptive immune responses. Science 346, 98–101. doi: 10.1126/science.1254536, PMID: 25170049 PMC4449618

[B29] Schenkel J. M. Fraser K. A. Masopust D. (2014b). Cutting edge: resident memory CD8 T cells occupy frontline niches in secondary lymphoid organs. J. Immunol. 192, 2961–2964. doi: 10.4049/jimmunol.1400003, PMID: 24600038 PMC3965619

[B30] Sullivan B. M. Juedes A. Szabo S. J. Von Herrath M. Glimcher L. H. (2003). Antigen-driven effector CD8 T cell function regulated by T-bet. Proc. Natl. Acad. Sci. U.S.A. 100, 15818–15823. doi: 10.1073/pnas.2636938100, PMID: 14673093 PMC307651

[B31] Surh C. D. Sprent J. (2008). Homeostasis of naive and memory T cells. Immunity 29, 848–862. doi: 10.1016/j.immuni.2008.11.002, PMID: 19100699

[B32] Teixeiro E. Daniels M. A. Hamilton S. E. Schrum A. G. Bragado R. Jameson S. C. . (2009). Different T cell receptor signals determine CD8+ memory versus effector development. Science 323, 502–505. doi: 10.1126/science.1163612, PMID: 19164748

[B33] Venkatesan P. (2024). The 2023 WHO World malaria report. Lancet Microbe 5, e214. doi: 10.1016/S2666-5247(24)00016-8, PMID: 38309283

[B34] Wikenheiser D. J. Stumhofer J. S. (2016). ICOS co-stimulation: friend or foe? Front. Immunol. 7, 304. doi: 10.3389/fimmu.2016.00304, PMID: 27559335 PMC4979228

[B35] Woon H. G. Braun A. Li J. Smith C. Edwards J. Sierro F. . (2016). Compartmentalization of total and virus-specific tissue-resident memory CD8+ T cells in human lymphoid organs. PLoS Pathog. 12, e1005799. doi: 10.1371/journal.ppat.1005799, PMID: 27540722 PMC4991796

[B36] Wu J. Madi A. Mieg A. Hotz-Wagenblatt A. Weisshaar N. Ma S. . (2020). T cell factor 1 suppresses CD103+ Lung tissue-resident memory T cell development. Cell Rep. 31, 107484. doi: 10.1016/j.celrep.2020.03.048, PMID: 32268106

[B37] Wu B. Zhang G. Guo Z. Wang G. Xu X. Li J. L. . (2021). The SKI proto-oncogene restrains the resident CD103(+)CD8(+) T cell response in viral clearance. Cell Mol. Immunol. 18, 2410–2421. doi: 10.1038/s41423-020-0495-7, PMID: 32612153 PMC8484360

[B38] Yang Y. Zhou Y. Wang J. Zhou Y. Watowich S. S. Kleinerman E. S. (2024). CD103(+) cDC1 dendritic cell vaccine therapy for osteosarcoma lung metastases. Cancers (Basel) 16 (19), 3251. doi: 10.3390/cancers16193251, PMID: 39409873 PMC11482638

[B39] Zarbock A. Kempf T. Wollert K. C. Vestweber D. (2012). Leukocyte integrin activation and deactivation: novel mechanisms of balancing inflammation. J. Mol. Med. (Berl) 90, 353–359. doi: 10.1007/s00109-011-0835-2, PMID: 22101734

[B40] Zhang X. Ouyang X. Xu Z. Chen J. Huang Q. Liu Y. . (2019). CD8+CD103+ iTregs inhibit chronic graft-versus-host disease with lupus nephritis by the increased expression of CD39. Mol. Ther. 27, 1963–1973. doi: 10.1016/j.ymthe.2019.07.014, PMID: 31402273 PMC6838901

[B41] Zirkel A. Lederer M. Stöhr N. Pazaitis N. Hüttelmaier S. (2013). IGF2BP1 promotes mesenchymal cell properties and migration of tumor-derived cells by enhancing the expression of LEF1 and SNAI2 (SLUG). Nucleic Acids Res. 41, 6618–6636. doi: 10.1093/nar/gkt410, PMID: 23677615 PMC3711427

