# Supplementary material for: Characteristics of CD103+CD8+ T cells in the spleen of Plasmodium yoelii NSM-infected mice
Source: Front Cell Infect Microbiol. 2025 Dec 11;15:1668438. doi: 10.3389/fcimb.2025.1668438 (PMC12738814; doi:10.3389/fcimb.2025.1668438)
Supplement: Supplementary file 1 [file Table1.docx]

**Supplementary Table 1. Antibodies used in the study**

| **Antibodies** | **Source** | **Origin** |
| --- | --- | --- |
| Anti-mouse CD45 APC (clone 30-F11) | Biolegend | America |
| Anti-mouse CD45 Alexa Fluor® 700 (clone A17A26) | Biolegend | America |
| eFluor 660 Fixable Viability Dye | Invitrogen | America |
| Anti-mouse CD3 FITC (clone 17A2) | Biolegend | America |
| Anti-mouse CD4 PerCP/Cyanine5.5 (clone RM4-4) | Biolegend | America |
| Anti-mouse CD8a APC/Cyanine7 (clone 53-6.7) | Biolegend | America |
| Anti-mouse NK1.1 PE/Cyanine7 (clone S17016D) | Biolegend | America |
| Anti-mouse TCRγ/δ APC (clone GL3) | Biolegend | America |
| Anti-mouse CD103 PE (clone 2E7) | Biolegend | America |
| Anti-mouse CD103 APC (clone 2E7) | BD PharMingen | America |
| Anti-mouse CD69 APC (clone H1.2F3) | BD PharMingen | America |
| Anti-mouse ICOS PE/Cyanine7 (clone C398.4A) | Biolegend | America |
| Anti-mouse PD-1 PE/Cyanine5 (clone 29F.1A12) | Biolegend | America |
| Anti-mouse CD62L APC (clone MEL-14) | Biolegend | America |
| Anti-mouse TIGIT PE/Cyanine7 (clone 1G9) | Biolegend | America |
| Anti-mouse/human CD44 Brilliant Violet 785™ (clone IM7) | Biolegend | America |
| Anti-mouse Ki-67 PE/Cyanine7 (clone 16A8) | Biolegend | America |
| Anti-mouse IFN-γ APC (clone XMG1.2) | Biolegend | America |
| Anti-mouse CD107a APC (clone 1D4B) | Biolegend | America |
| Anti-human/mouse Granzyme B PE/Cyanine7 (clone QA16A02) | Biolegend | America |
| Anti-mouse Perforin PE (clone S16009A) | Biolegend | America |
| Alexa Fluor 488 Conjugate LEF1 (clone C12A5) | Cell Signaling | America |

**Supplementary Table 2. Prime for gene detected in this study.**

| **Gene** | **Forward primer** | **Reversed primer** | |
| --- | --- | --- | --- |
| ***Itgae*** | GGGTCCTACTTTGGCTCTGT | | GTGTGTGTGCCAAGGAGAAG |
| ***Lef1*** | GCCACCGATGAGATGATCCC | | TTGATGTCGGCTAAGTCGCC |
| ***Tcf7*** | AACTGGCCCGCAAGGAAAG | | CTCCGGGTAAGTACCGAATGC |
| ***Tgfb1*** | CTTCAATACGTCAGACATTCGG | | GTAACGCCAGGAATTGTTGCTA |
| ***β-actin*** | CCGTAAAGACCTCTATGCCAAC | | GGGTGTAAAACGCAGCTCAGTA |

**Supplementary Table 3. Alterations in the abundance and composition of splenic CD45⁺ immune cell clusters following infection.**

| **Cluster** | **Normal** | **12dpi** |
| --- | --- | --- |
| B cells | 4527 (18.83%) | 5088 (50.89%) |
| CD4 T cells | 3036 (26.45%) | 1431 (14.31%) |
| CD8 T cells | 2750 (23.59%) | 860 (8.60%) |
| DCs | 41 (0.35%) | 101 (1.01%) |
| Erythrocyte | 5 (0.04%) | 138 (1.38%) |
| Macrophages | 250 (2.14%) | 187 (1.87%) |
| NK cells | 562 (48.22%) | 47 (0.47%) |
| NKT cells/ gdT cells | 15 (0.13%) | 238 (2.38%) |
| Neutrophils | 231 (1.98%) | 504 (5.04%) |
| Plasma cells | 50 (0.43%) | 538 (5.38%) |
| Proliferating B cells | 95 (0.82%) | 471 (4.71%) |
| Proliferating T cells | 94 (0.81%) | 395 (3.95%) |
| Total | 11656 | 9998 |


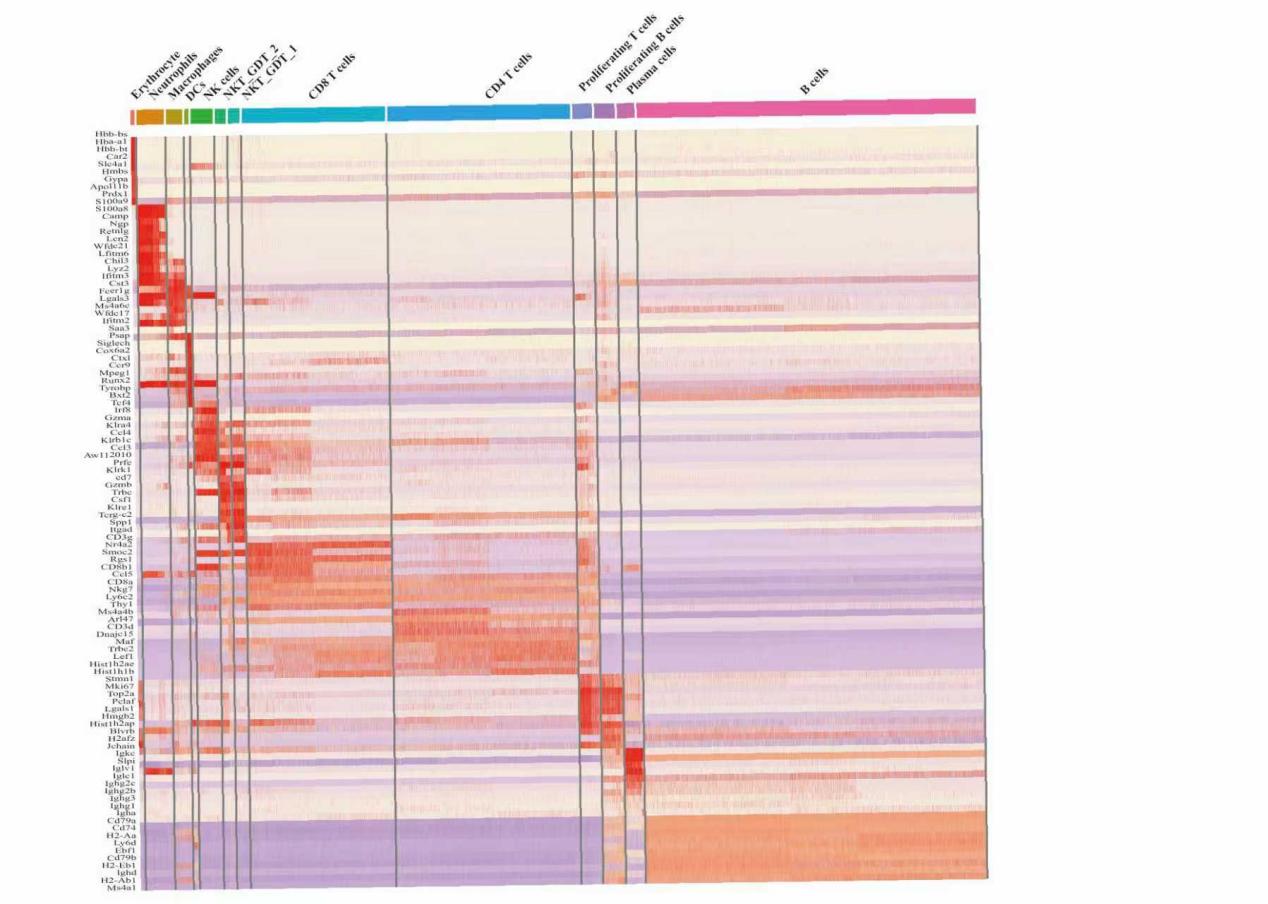


**Figure S1: Single-cell RNA sequencing and cluster annotation of splenic CD45+ immune cells.**

Heat map for cell type annotation of the 12 clusters based on the expression of key lineage-defining marker genes. The color intensity represents the average scaled expression level across expressing cells.


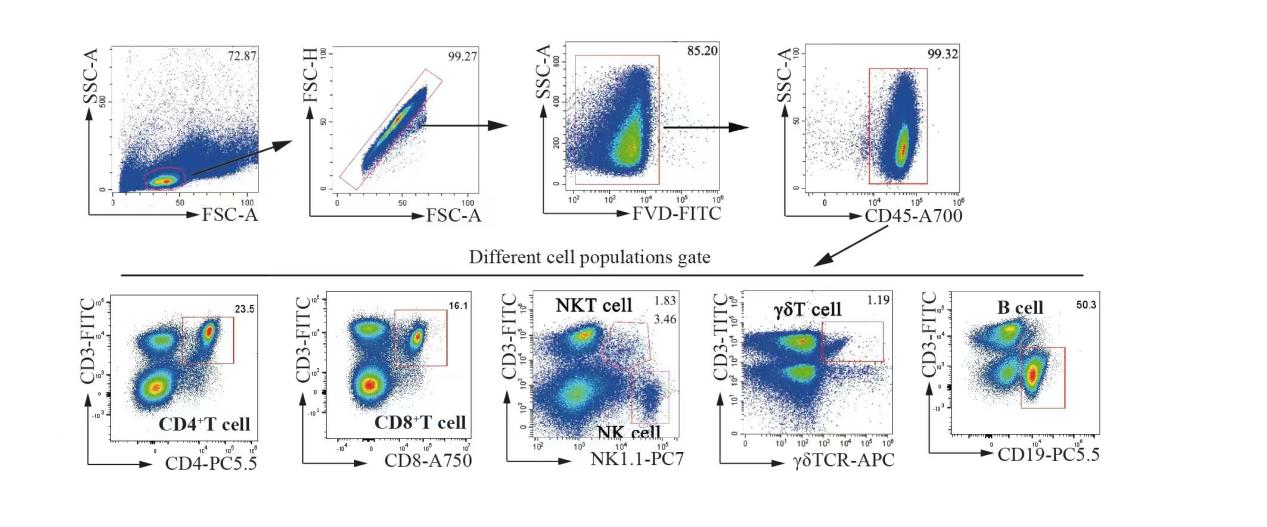


**Figure S2: Expression of CD103 on various lymphocyte subsets following Plasmodium infection.**

Related to Figure 1, the gating strategy to select total lymphocytes, followed by CD4^+^ T, CD8^+^ T, CD3^-^NK1.1^+^ NK cells, CD3^+^NK1.1^+^ NKT cells, CD3^+^γδTCR^+^ γδT cells, and CD3^-^CD19^+^ B cells.

**
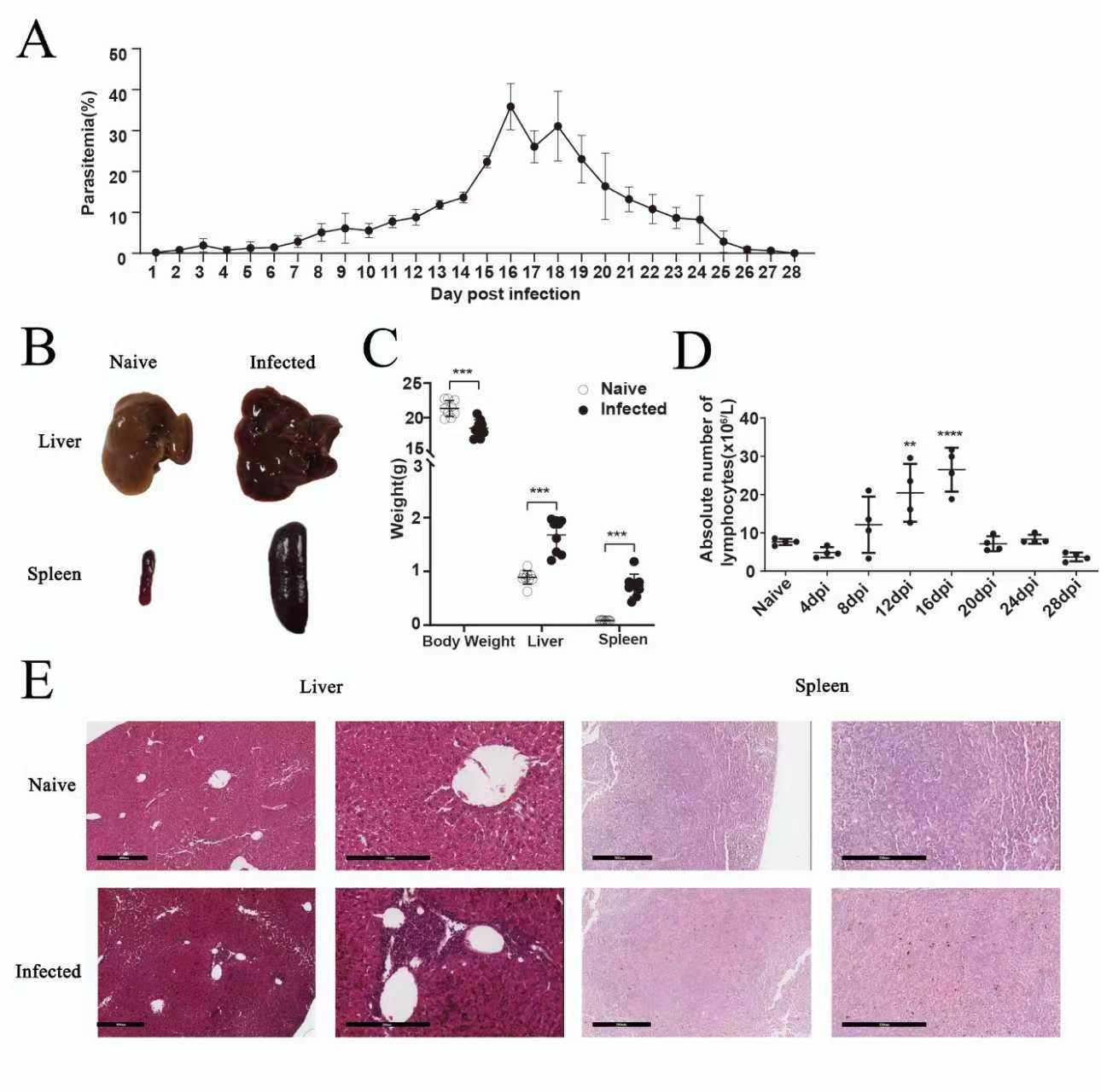
**

**Figure S3: Systemic inflammatory changes induced by Plasmodium infection**

Thirty-two 6-8-week-old female C57BL/6 mice were intraperitoneally infected with *Plasmodium yoelii* NSM. (A) Parasitemia was monitored by daily tail vein blood sampling (3 randomly selected mice per day, sequentially). (B, C) Photography and weight of liver and spleen picked out from both naïve and infected mice (12-16 dpi). (D) Every 4 days post-infection, three mice were euthanized to assess peripheral blood RBC counts and the splenic lymphocyte counts (from single-cell suspensions). (E)Tissue samples were taken from the liver and spleen of both naïve and infected mice (12-16 dpi), and paraffin sections were prepared. HE staining was done, and the pathological changes in the tissues of infected mice were shown. Data from multiple replicates (3 mice per group) are shown as mean ± SEM. Statistical significance: Student’s t-test; *ns* *p*> 0.05, **p*< 0.05, ***p*< 0.01, ****p*< 0.001, *****p*< 0.0001.


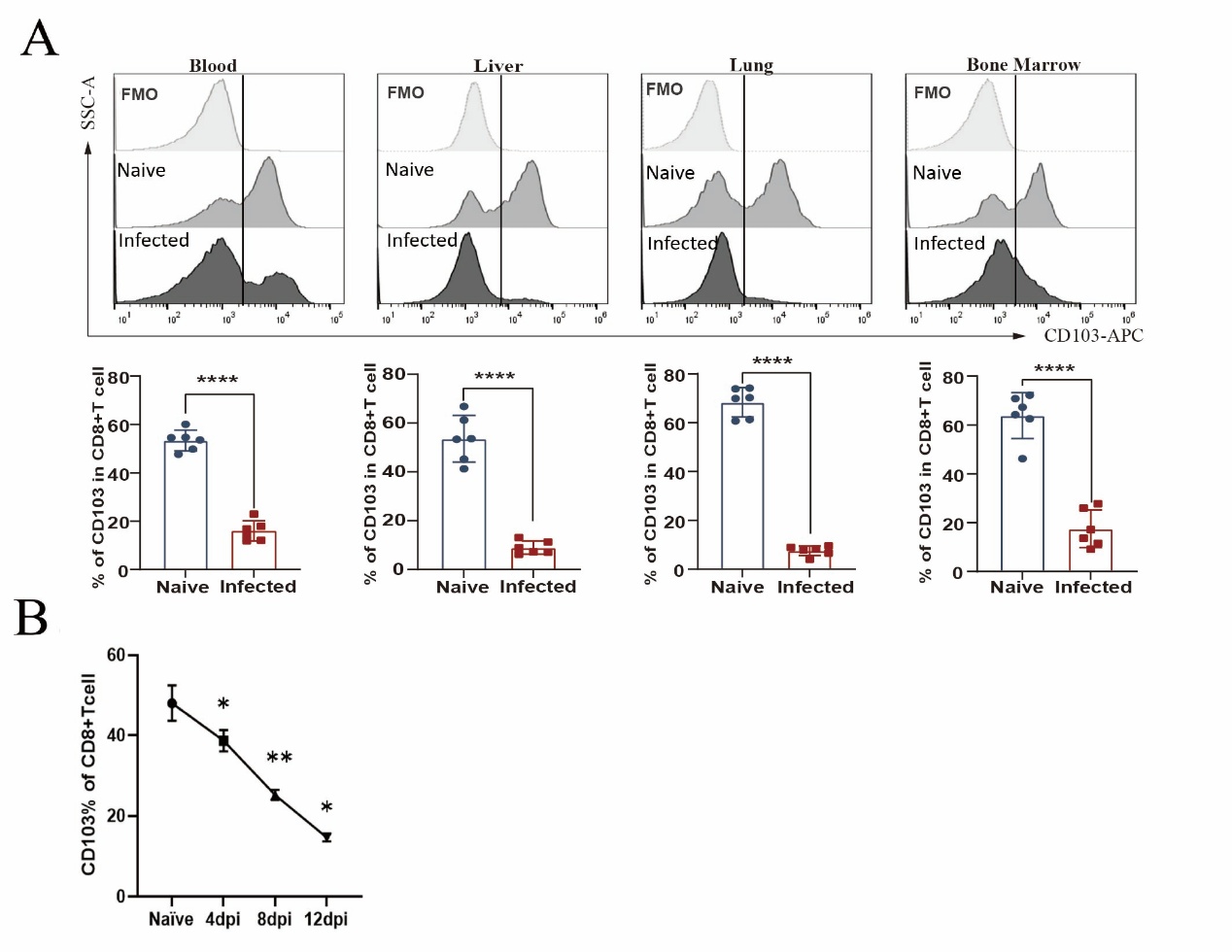


**Figure S4: Concentrated expression of CD103 molecules on CD8+ T cells in different tissues**

Lymphocytes from peripheral blood, lung, liver, and bone marrow were extracted to analyze the difference in CD103 expression in CD8^+^ T cells before and after infection. (A) Longitudinal analysis of CD103 expression from blood CD8^+^ T cells after infection. The peripheral blood of normal mice and mice infected with Plasmodium for 4 days, 8 days, and 12 days was separated. Peripheral blood mononuclear cells were isolated. FACS detected the percentage of CD103+ cells in CD8+ T cells. (B) The experiment was repeated 2 times. Statistical results are shown as mean ± SEM, with statistical significance determined by *Student's t*-test, not significant (ns), *p* > 0.05, **p* < 0.05, ***p* < 0.01, ****p* < 0.001, ****p* < 0.0001.


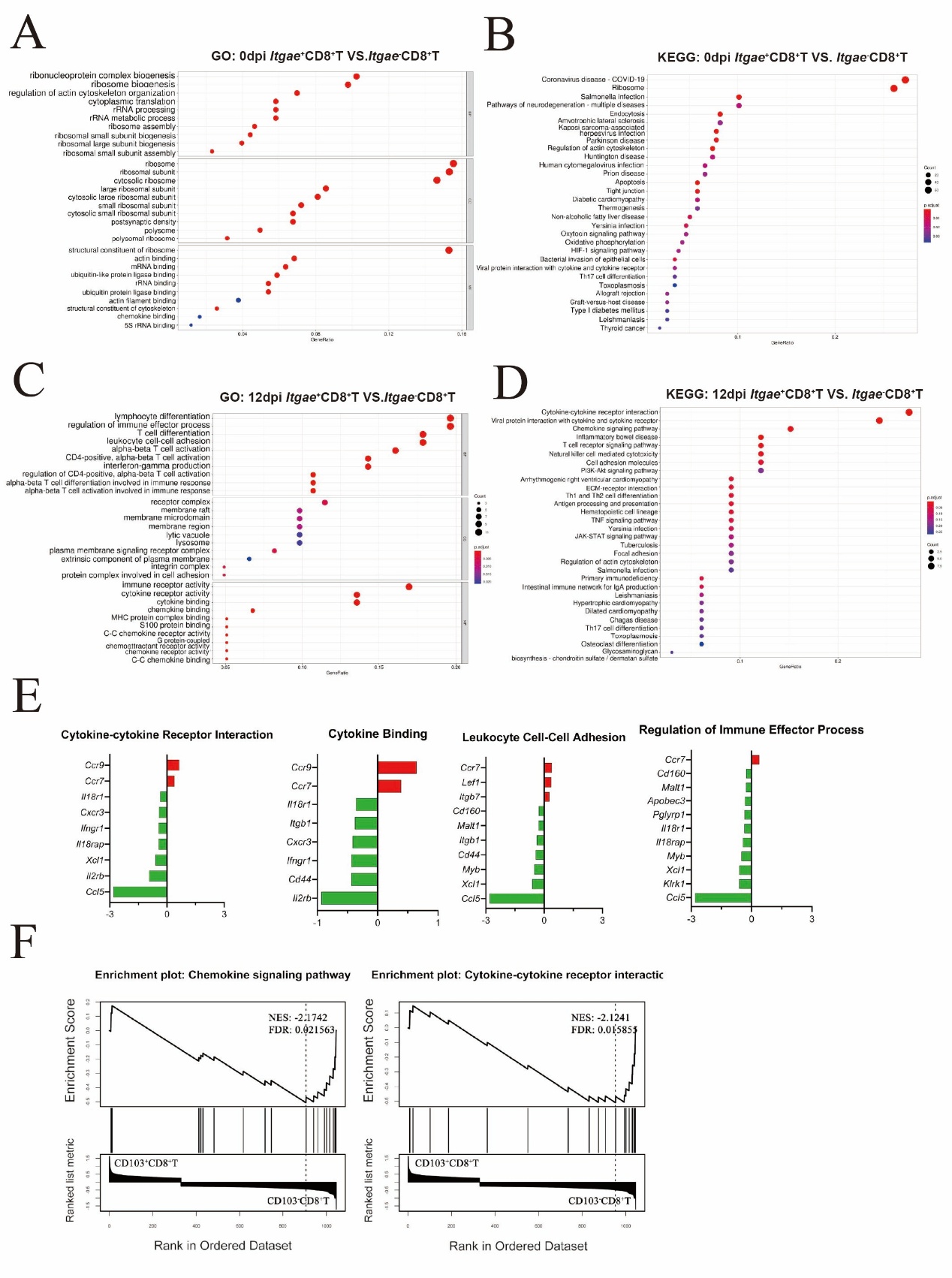


**Figure S5: Bioinformatics analysis between*Itgae*^-/+^ CD8^+^T cells in naïve and infected stage.**

(A-D)GO enrichment analysis and KEGG analysis of differentially expressed genes between *Itgae*^+^ cells and *Itgae*^-^ cells of spleen CD8^+^ T cells in naïve (0 dpi) and infected (12 dpi) mice. (E) The partial enrichment pathway results are displayed. (F)Differentially expressed genes from the infected group were analyzed using the GSEA gene set, which includes "Chemokine signaling pathway" and "Cytokine-cytokine receptor interaction".
